# Human epidermal growth factor receptor 2 expression in women with uterine cervix adenocarcinoma from Appalachian Kentucky

**DOI:** 10.3389/fonc.2023.948348

**Published:** 2023-01-24

**Authors:** Charles A. Kunos, Denise Fabian, Dava W. Piecoro, Dana Napier, Rachel W. Miller, Frederick R. Ueland

**Affiliations:** ^1^ Department of Radiation Medicine, University of Kentucky, Lexington, KY, United States; ^2^ Department of Pathology and Laboratory Medicine, University of Kentucky, Lexington, KY, United States; ^3^ Biospecimen Procurement & Translational Pathology, University of Kentucky, Lexington, KY, United States; ^4^ Department of Obstetrics & Gynecology, Division of Gynecologic Oncology, University of Kentucky, Lexington, KY, United States

**Keywords:** uterine cervical cancer, uterine cervical adenocarcinoma, HER2/neu, radiopharmaceutical, triage

## Abstract

**Introduction:**

High-risk human epidermal growth factor receptor 2 (HER2)-positive adenocarcinomas associate with early recurrence and death, prompting consideration of novel radiotherapeutic options like a trastuzumab-linked thorium-227 alpha-particle emitting radionuclide.

**Methods:**

We conducted a retrospective pilot biomarker study of uterine cervix cancers among patients in Appalachian Kentucky, to characterize an exploitable triage biomarker like HER2 expression before starting a prospective phase 0 trial.

**Results:**

Most (60%) adenocarcinomas showed HER2 cell-surface overexpression, whereas squamous cell carcinomas (4%) did not do so.

**Discussion:**

Further validation tests of HER2 expression as a triage biomarker for radiopharmaceutical selection are warranted.

## Introduction

Uterine cervix cancer incidence dropped by 54 percent from the early-1970s to the early-2000s (1), mainly due to the common medical practice of cancer screening with the combined Papanicolaou cytological examination and reflex human papillomavirus test (2, 3). But, the incidence of this disease has remained unchanged over the last decade (4). The Commonwealth of Kentucky has risen to first among United States (U.S.) states in uterine cervix cancer incidence, and its Appalachian region accounts for this astonishing health burden (12.6 per 100,000 [versus 7.7 per 100,000 in the total U.S.], age-adjusted to the 2000 U.S. standard population, 4, 5). In addition, distinctions in uterine cervix cancer clinical presentation and treatment are found among women of minority groups or when impoverished in Appalachia due to inadequate access to cancer screening programs (6).

High-risk non-surgical advanced-stage adenocarcinomas or adenosquamous carcinomas of the uterine cervix are frequently associated with early recurrence and high mortality (7). Platinum-based radiochemotherapy is a preferred initial treatment approach (8–10). A four-drug regimen that includes pembrolizumab has emerged in the first-line for those with persistent, recurrent, or metastatic uterine cervix cancer (11).

Uterine cervix cancers are clinically diverse tumors, with tumor bulk associating with a more indolent or more aggressive course (prognostic factor) as well as correlating with a very good or very poor response (predictive factor). For example, in a phase III clinical trial of cisplatin-radiotherapy for bulky or barrel-shaped uterine cervix cancer, the size of tumor predicted for poorer response and survival (8). Molecular drivers of the bulky or barrel-shaped phenotype were not investigated. For some investigators, overactivity or overexpression of the human epidermal growth factor receptor 2 (HER2) might be causally involved as a molecular alteration in the bulky or barrel-shaped phenotype. HER2 stimulates tumorigenesis by expanding tumor cell number, deregulating cell cycle progression, disrupting cell adhesion and polarity, and promoting an invasive phenotype. Targeted therapies against HER2 on cancer cells are desirable.

Trastuzumab (Herceptin, Genentech, Inc., South San Francisco, California), an anti-HER2 monoclonal antibody, has been shown to have antitumor activity and a range of mostly low-grade toxic effects in women with metastatic HER2+ breast cancer (12, 13). Trastuzumab has not been tested in trials for women with uterine cervix cancer. But, HER2 inhibition may enhance radiosensitivity through a reduced S-phase cell cycle fraction (14). Preclinical results have shown that a HER2 molecularly-targeted thorium-227 trastuzumab conjugate alpha-particle emitting radiopharmaceutical has promising antitumor activity without undue toxic effects (15, 16). Thus, there is an opportunity for early clinical development of a HER2-targeting radiopharmaceutical in women with persistent, recurrent, or metastatic uterine cervix, perhaps in a trial enriched in design for adenocarcinomas or adenosquamous carcinomas (17). Indeed, in the National Cancer Institute Cancer Therapy Evaluation Program clinical development path, a preferred and leveraged programmatic strategy engages a biomarker-driven enrichment trial design (18, 19).

This pilot study evaluates the feasibility of identifying biomarker enrichment retrospectively, acquiring tumor for HER2 expression as a sufficiently discriminating biomarker in adenocarcinomas of the uterine cervix against other cell types, prior to launching a phase 0 trial investigating state-of-the-art molecular profiles as possible explanations for treatment response (NCT05462951). A second goal was to assess the usefulness of uterine cervix cancer cell type and tumor size as enrichment trial-enabling eligibility criteria for early-phase radiopharmaceutical studies. The study also discusses uterine cervix cancer screening and patient socioeconomic factors that are likely to impact operationalization of triage HER2 immunohistochemistry prior to radiopharmaceutical selection intending to treat persistent, recurrent, or metastatic uterine cervix cancer.

## Materials and methods

### Study population

The University of Kentucky Markey Cancer Center (MCC) compiles information for uterine cervix cancer diagnoses among residents of the state, inclusive of demographic, clinical, and diagnostic data as well as cancer treatment. The MCC routinely connects its data to that of the Surveillance, Epidemiology, and End Results (SEER) Kentucky Cancer Registry (KCR, ref. 5) to discover any omitted uterine cervix cancer diagnoses and to obtain additional data on prior cases. Each year MCC and KCR match possible unreported uterine cervix cancer cases with Kentucky death certificates that designate cancer diagnoses. KCR additionally matches its database to the Social Security Administration database, the Centers for Medicare and Medicaid Services database, and the National Death Index for data on vital status. MCC and its affiliate community oncology practices provide cancer care for a rural agricultural and urban manufacturing region inclusive of 4.5 million persons in central and eastern Kentucky. In the present study, a deidentified MCC study population of 308 randomly-sampled women aged 18 years or older diagnosed with invasive uterine cervix cancer were reviewed among the 4,186 KCR-registered invasive uterine cervix cancer patients (January 2001 to March 2022). The total study population had a median follow-up of 33 months and 58 percent were alive at the time of data cut-off (March 31, 2022). Usual for the MCC catchment, 96 percent were of Caucasian (white) race and 63 percent were active smokers. The Institutional Review Board at the University of Kentucky (Lexington, Kentucky, #69443) approved this retrospective study.

### Exposures and assessments

MCC collects data on initial cancer treatment, including total extrafascial hysterectomy with bilateral salpingo-oophorectomy with or without lymphadenectomy (hereafter, surgery) only; surgery plus radiotherapy; surgery plus chemotherapy; radiochemotherapy only; radiochemotherapy plus surgery; or chemotherapy only. Radiotherapy involved conventional external-beam irradiation or intensity-modulated radiation therapy delivered five days a week. Either conventional low-dose-rate or high-dose-rate intracavitary or interstitial brachytherapy was prescribed to point A or an individualized contoured tumor volume. Radiochemotherapy added cisplatin (40 mg m^-2^) chemotherapy, not to exceed 70 mg total per week, to the radiotherapy once a week for a maximum of six cycles. Chemotherapy involved paclitaxel (175 mg m^-2^) and either a choice of cisplatin (50 mg m^-2^) or carboplatin (area under the concentration–time curve, 5 mg ml^-2^ min^-1^) every three weeks. It may also have included bevacizumab (15 mg kg^-1^) every three weeks at the treating physician’s discretion. In general, follow-up for disease status and survival was scheduled every three months for the first two years with cancer care providers, then every six months for years three to five, and then annually. Surveillance imaging was obtained when disease symptoms emerged or at the treating physician’s discretion.

### Ascertainment of uterine cervix cancer screening and health insurance coverage

The use of the combined Papanicolaou cytological examination and reflex human papillomavirus test was ascertained by MCC through database linkage to the University of Kentucky HealthCare electronic medical record using the International Classification of Diseases-10 (ICD-10) codes for uterine cervix cancer (C53). Precursor lesions of either high-grade squamous intraepithelial lesion (HSIL) or adenocarcinoma *in situ* (AIS) were catalogued and linked to the later cell type of invasive cancer (**Table 1**). In this analysis, only invasive uterine cervix cancer cell types of squamous, adenosquamous, and adenocarcinoma were included. Health insurance coverage was abstracted in the same database linkage using terminology determined by the United States Census Bureau (20). Individuals were considered uninsured if they did not have health insurance coverage as of March of the prior calendar year at diagnosis (**Table 2**).

**Table 1:** Prevalence of papanicolaou screening, human papillomavirus genotyping, and high-grade squamous intraepithelial lesion or adenocarcinoma *in situ* or worse in the studied women with uterine cervix cancer from Kentucky: 2001 to 2021.

Variable	Appalachian Kentucky	Non-Appalachian Kentucky	*P*
Total in uterine cervix cancer population^1^	208 (68)	100 (32)	
Median age in years (25%-75% quartile)	53 (43-63)	51 (42-60)	0.23
Prevalence of Papanicolaou cytology, any	68 (33)	52 (52)	0.001
Prevalence of Papanicolaou cytology, <3 years^2^	37 (18)	40 (40)	<0.001
Prevalence of HPV genotyping	15 (7)	21 (21)	<0.001
Prevalence of any high-risk HPV^3^	13 (87)	20 (95)	0.36
Prevalence of HPV 16^3^	8 (53)	7 (33)	0.23
Prevalence of HPV 18^3^	2 (13)	6 (29)	0.28
Prevalence of HPV 31, 33, 35, 45, 52, or 58^3^	3 (20)	7 (33)	0.38
Prevalence of HSIL^4^	27 (40)	27 (52)	0.18
Prevalence of squamous cell carcinoma	143 (69)	68 (68)	0.89
Prevalence of AIS^4^	8 (12)	4 (8)	0.46
Prevalence of adenosquamous cell carcinoma	11 (5)	10 (10)	0.12
Prevalence of adenocarcinoma	54 (26)	22 (22)	0.45

AIS, adenocarcinoma in situ; HPV, human papillomavirus; HSIL, high-grade squamous intraepithelial lesion.

^1^ Numbers in parentheses are the proportion (%) of the total unless otherwise indicated.

^2^ Indicates Papanicolaou smear within three years of cancer diagnosis.

^3^ Proportion of those women undergoing HPV genotyping.

^4^ Proportion of those women undergoing any Papanicolaou smear.

**Table 2:** Number of women in Kentucky with uterine cervix cancer by health insurance coverage: 2001 to 2021.

Coverage Type	Appalachian Kentucky	Non-Appalachian Kentucky	*P*
Total in uterine cervix cancer population	208 (68)	100 (32)	
Any health plan^1^	182 (88)	88 (88)	0.90
Any private plan^2^	20 (10)	20 (20)	0.01
Employment-based	16 (8)	15 (15)	0.05
Direct-purchase	4 (2)	5 (5)	0.03
Any public plan^3^	162 (78)	68 (68)	0.06
Medicare	56 (27)	18 (18)	0.09
Medicaid	103 (50)	49(49)	0.93
VA or CHAMPVA	3 (1)	1 (1)	0.75
Uninsured	26 (12)	12 (12)	0.90

^1^ The number of women (%) by types of coverage are not mutually exclusive; women can be covered by more than one coverage plan.

^2^ Private health coverage includes coverage through an employer or union, or coverage purchased directly.

^3^ Public health coverage includes, Medicare, Medicaid, CHAMPVA (Civilian Health and Medical Program of the Department of Veterans Affairs), and care provided by the Department of Veterans Affairs (VA) or the military.

### Immunohistochemistry

Uterine cervix cancers were sectioned at four micrometers and mounted onto positively charged slides which were baked at 58 °C overnight. Slides were stained using a Ventana Discovery Ultra Autostainer (Ventana Medical Systems, Tucson, Arizona) per the manufacturer’s instruction. Deparaffinization was performed on the instrument, followed by washing with Ventana Reaction Buffer (Roche 950-300, Basel, Switzerland), which was used for all subsequent washes. Slides then underwent on-board antigen retrieval with CC1 buffer (Roche) using mild conditions (95 °C, 32 minutes). Slides were washed again and incubated with peroxidase quench for four minutes at 37 °C (Roche ChromoMap DAB Kit, 760-159), followed by washing and incubation with anti-Her2/Neu antibody (Clone 4B5, Roche 790-2991) for 20 minutes at 37 °C. After washing, slides were incubated with OmniMap anti-Rabbit-HRP polymer (Roche 760-4311) at 37 °C for 20 minutes. Slides were again washed, and the signal was visualized by incubation with ChromoMap DAB (Roche 760-159) at 37 °C. Slides were removed from the Autostainer and washed before manually counterstaining in Meyer’s hematoxylin (Fisher H345-25) for five minutes at room temperature, followed by distilled water wash and bluing in 1% ammonia water (Fisher Scientific 458690025, Waltham, Massachusetts). Slides were dehydrated stepwise through ethanol, then cleared in two exchanges of xylene, and mounted with glass coverslips and Cytoseal mounting media (Andwin Scientific 48212187, Tryon, North Carolina).

### Microscopy

**Figure 1:**
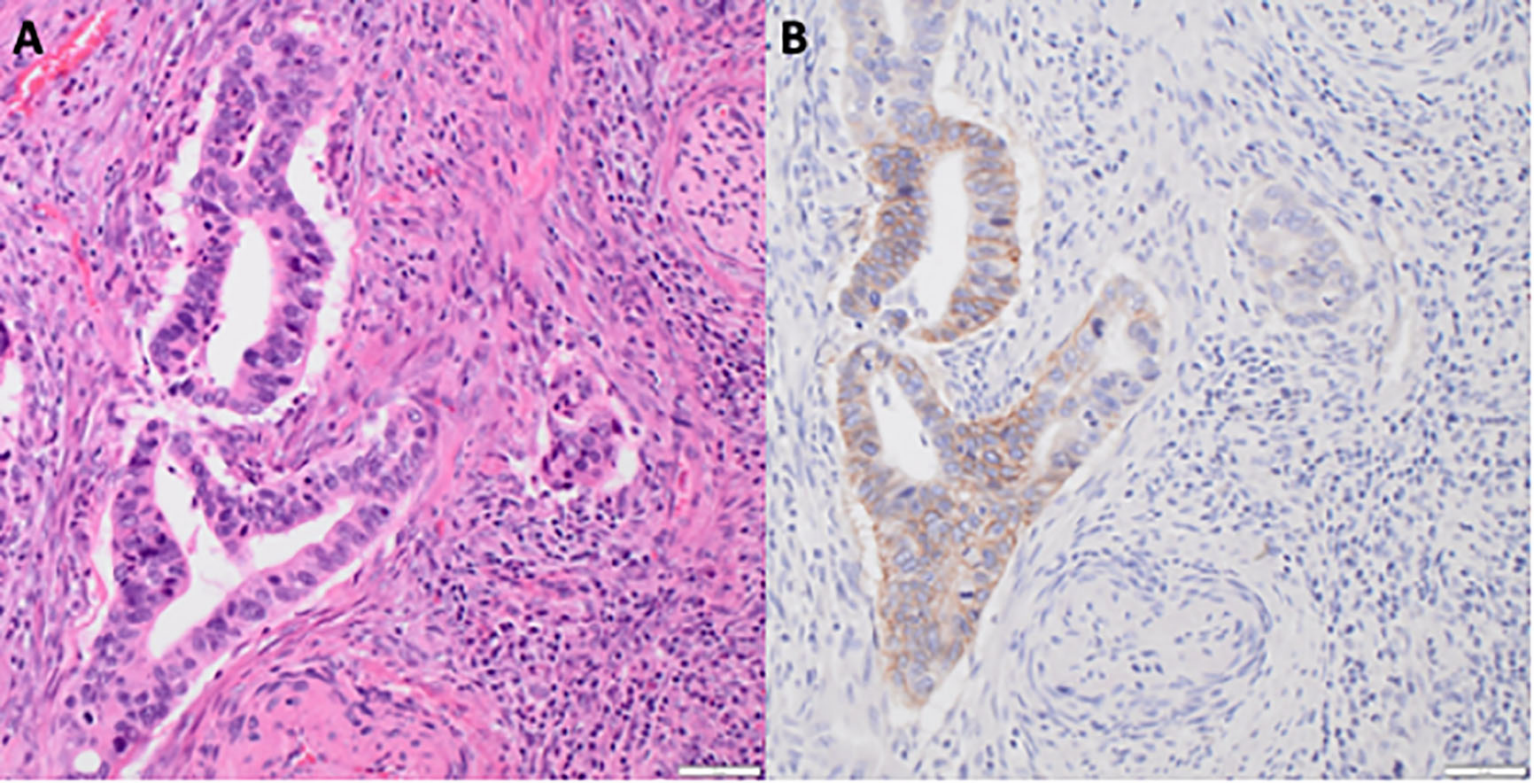
HER2 immunoreactivity in uterine cervix adenocarcinoma. **(A)** Hematoxylin and eosin staining of uterine cervix adenocarcinoma at ×20 magnification with scale bar. **(B)** HER2 brown staining of uterine cervix adenocarcinoma at ×20 magnification with scale bar.

Individual slides of uterine cervix cancer tissues were viewed on an inverted microscope (Olympus, Center Valley, Pennsylvania) at ×20 magnification (**Figure 1**). One histopathologist blinded to treatment outcome scored the brown staining intensity of HER2 using a similar reporting format to that used for reporting results of HER2 testing for breast cancer (21). Digital images of uterine cervix cancer slides with scale bars were acquired using an Olympus DP23 camera with Olympus cellSens Standard 3.2 image analysis software (Olympus) at ×20 magnification.

### Statistical analyses

The primary endpoint of this retrospective analysis was positive HER2 immunohistochemistry defined arbitrarily as 1+-3+ HER2 antibody brown cell-surface stain at the time of microscopy because any level of expression would permit a conjugated radiopharmaceutical access to target. Secondary endpoints involved overall survival in the combined adenocarcinoma/adenosquamous uterine cervix cancer population and in those with a minimum clinical tumor diameter of six centimeters or more. Screening and health insurance coverage were evaluated in all patients as indicators for the ease of implementing an immunohistochemical triage assay for radiopharmaceutical selection.

Immunohistochemistry was assessed on 29 uterine cervix cancer patients who had previously consented to the future scientific study of their tumors and had their samples stored at the Biospecimen Procurement & Translational Pathology Shared Resource Facility at the University of Kentucky Markey Cancer Center. Our primary hypothesis tested whether adenocarcinoma/adenosquamous cell types had higher immunohistochemical expression of HER2 than squamous cell types. The Fisher exact test computed *P* values for such associations (Microsoft Corporation, 2019. Microsoft Excel). In compiling the samples, we made an *a priori* decision to include any patient with available uterine cervix cancer tissue without a duplicate sample. This limits sample size and allows only exploratory analysis of HER2 immunohistochemistry by cell type.

The Kaplan-Meier product-limit method with 95 percent confidence intervals (CI) for survival was computed using statistical software (Microsoft Excel). The log-rank test was used for comparisons. Other descriptive statistics, Pearson’s chi-square test for categorical variables, or Student’s t-test for continuous variables were computed using statistical software (Microsoft Excel). For all, a *P* value less than 0.05 (two-sided) indicated statistical significance. Data are available from the authors under restriction and upon reasonable request, with permission from MCC.

## Results

### Study population

The 308 women had the following cell types on diagnostic histopathology—211 (69%) squamous cell carcinomas, 76 (25%) adenocarcinomas, and 21 (7%) adenosquamous carcinomas. The median age at diagnosis was 53 years (25%-75% quartile range, 43-61 years). A total of 208 (68%) patients lived in Appalachian counties of eastern Kentucky and 100 (32%) patients resided elsewhere in central Kentucky. There were no differences in clinical factors among Appalachian and non-Appalachian residents. The median clinical tumor diameter (gross tumor only) was five centimeters (25%-75% quartile range, 4-6 centimeters); 86 (28%) had a minimum clinical tumor diameter of six centimeters or more. The stage at diagnosis was 27 percent localized (primary site only), 54 percent regional (spread beyond the primary site or spread to pelvic lymph nodes), and 19 percent distant (metastasized). There were no significant differences among Appalachian and non-Appalachian residents with regard to the following histopathological factors—cell type, depth of invasion, grade, angiolymphatic invasion, or positive pelvic adenopathy. Treatment involved surgery only in 19 (6%) patients, surgery plus radiotherapy in seven (2%) patients, radiochemotherapy only in 231 (75%) patients, and radiochemotherapy plus surgery in 51 (17%) patients.

### Uterine cervix cancer HER2 immunohistochemistry

HER2 overexpression and/or *ErbB2* gene amplification or aberrant transcription results in an up to a 100-fold increase in cancer cell surface HER2 and consequently drives HER2-mediated tumorigenesis. To evaluate whether cell surface HER2 overexpression holds in *in situ* uterine cervix cancers, we evaluated the pattern of cell-surface expression of HER2 in uterine cervix cancers. HER2 antibody staining intensity was 1+ or 2+ in the majority (3/5 [60%]) of the adenocarcinoma uterine cervix cancers (**Figure 1**, *P* = 0.001). HER2 antibody staining intensity was positive in almost none (1/24 [4%]) of the squamous cell uterine cervix cancers.

### Uterine cervix cancer survival

With 130 (42%) observed deaths, Kaplan-Meier estimates of the proportion of women at 36 months who were alive were 68 percent (95% CI, 57-78%) in the adenocarcinoma-adenosquamous carcinoma group and 78 percent (95% CI, 73-85%) in the squamous cell carcinoma group. The proportion of women at 60 months who were alive was 59 percent (95% CI, 48-70%) in the adenocarcinoma-adenosquamous carcinoma group and 71 percent (95% CI, 63-78%) in the squamous cell carcinoma group. A trend for worse survival in the adenocarcinoma-adenosquamous carcinoma group does not reach statistical significance (*P* = 0.09).

Regional disease control and survival in patients with large (≥6 cm) uterine cervix cancers are poorer than in patients with smaller tumors, whether treated by surgery, radiation, or chemotherapy alone or in combination. To test whether this observation applies to our uterine cervix cancer population, we compared survival among patients with tumors measuring a minimum clinical tumor diameter of six centimeters or more versus not. Kaplan-Meier estimates of the percentage of women alive at 36 months were 58 percent (95% CI, 45-69%) in the six centimeters or more group and 82 percent (95% CI, 76-87%) in the less than six centimeters group. The percentage of women at 60 months who were alive was 47 percent (95% CI, 34-59%) in the six centimeters or more group and 75 percent (95% CI, 67-81%) in the less than six centimeters group. This is statistically significant (*P* = 0.002).

### Uterine cervix cancer screening and health insurance coverage

The prevalence of cancer screening using the combined Papanicolaou cytological examination and reflex human papillomavirus test is found in **Table 1**. Among Kentucky residents who developed invasive uterine cervix cancer under study here, the act of undergoing any Papanicolaou cytological examination during their lifetime was infrequent (39%), and doing so within three years of their diagnosis, was uncommon (25%, **Table 1**). Only 12 percent of women had any human papillomavirus test (**Table 1**). There were significant differences in screening, with the procedure done more frequently among women residing in non-Appalachian Kentucky than those living elsewhere in Appalachia (*P* = 0.001). The proportions of human papillomavirus genotypes 16, or 18, or one of 31, 33, 35, 45, 52, or 58 were 45 percent, 25 percent, and 30 percent, respectively (**Table 1**). Detecting a high-grade squamous intraepithelial lesion (26%) before a diagnosis of squamous cell carcinoma or detecting an adenocarcinoma *in situ* lesion (12%) before a diagnosis of adenocarcinoma or adenosquamous carcinoma was also uncommon (**Table 1**).

The number of women with health care coverage at the time of treatment is listed in **Table 2**. The proportion of uninsured or those having Medicaid assigned at the time of treatment was 62 percent. The proportion of uninsured or Medicaid recipients was not different among Appalachian and non-Appalachian residents (**Table 2**).

## Discussion

In this retrospective study involving women with uterine cervix cancer residing in or near Appalachian Kentucky, a significantly higher proportion (60%) of women with adenocarcinomas than squamous cell carcinomas (4%) had positive HER2 immunohistochemistry. This finding differs from the results of a Mexico City study, which found positive HER2 staining only in a single patient (1 [3%] of 35) with squamous cell carcinoma of the uterine cervix (22); the inconsistent results may be related to different disease stages, HER2 assays, lack of adenocarcinoma or adenosquamous cell types, or all of these factors. Prospective analyses of molecular biomarkers that might aid the selection of a targeted radiopharmaceutical are planned in our phase 0 uterine cervix cancer patient trial (NCT05462951).

The present results fit into the context of a previous treatment trial studying a pan-HER tyrosine kinase inhibitor in patients with metastatic uterine cervix cancer. In the phase II SUMMIT study of neratinib for recurrent metastatic uterine cervix cancer (23), eligible patients had a likely pathogenic mutation in *ErbB2.* Thirteen (81%) of 16 patients had adenocarcinoma (23). The most frequent *ErbB2* genotype variant was a hotspot S310F/Y mutation, occurring in 10 (63%) of the 16 patients. The overall response rate of neratinib was 19 percent (3 of 16); all occurred in women with adenocarcinoma uterine cervix cancer. Otherwise, there is only a single case report of a woman with metastatic adenocarcinoma of the uterine cervix who received the combination of trastuzumab-pertuzumab treatment repeated every 28 days (24). She had a durable 27-month disease response. Taken together and with the observation that trastuzumab reduces the radioresistant S-phase fraction (14), these results suggest a HER2-targeted radiopharmaceutical, such as the alpha-particle emitting thorium-227 trastuzumab conjugate, might have clinical benefit in patients with HER2-positive adenocarcinoma or adenosquamous carcinoma of the uterine cervix.

Uterine cervix cancer may be prevented through rigorous programs operationalizing the administration of vaccines for this disease and screens for precursor lesions with proper follow-up and intervention. Uterine cervix cancer screens, like those for breast and colon cancer, might detect malignancy early and lessen the risk of death from disease. And like other screens, abnormal cytology and human papillomavirus tests necessitate patient triage to colposcopy, biopsy, and treatment of premalignant lesions. Despite a lack of reproducibility and subjectivity of result interpretation, cytological evaluation remains the preferred screening practice. Barriers such as lack of public or private insurance coverage of these uterine cervix cancer screens remain troublesome in poorly resourced regions like Appalachian Kentucky. A more sophisticated analysis of health insurance coverage was not possible in this study.

Study limitations include that we did not immunohistochemically characterize more than five adenocarcinomas/adenosquamous carcinomas of the uterine cervix. We did not have the opportunity to assess more tumors in this pilot study due its retrospective design and lack of further uterine cervix cancer tissue in the biospecimen and translational pathology repository at the University of Kentucky. For a true comparison, pathological features of adenocarcinoma cases, such as size, grade, and line of treatment, would need to be matched to those of squamous cell carcinoma cases. This level of effort could not be justified unless the pilot study offered possible distinction in HER2 expression and in patient survival. An additional limitation is that we cannot link HER2 expression directly to tumor bulk and associate any durable treatment response as these data were unavailable. We also included patient tumors who underwent multiagent and multimodality treatment, which may confound HER2 expression and patient outcome. Acceptance of such cases relied on an *a priori* decision to evaluate all uterine cervix cancer biospecimens whether patients completed therapy or not. To overcome these limitations, a prospective phase 0 study has begun patient accrual (NCT05462951).

Our team believes it is important to use molecular triage when selecting a radiopharmaceutical to treat persistent, recurrent, or metastatic uterine cervix cancer (17, 18). Molecular triage would aid the design of early phase trials to maximize possible clinical benefit and limit exposure to potential toxicity, and it could have a substantial impact on the overall clinical development of the radiopharmaceutical. Here, we found a possibly discriminating cell-surface biomarker of HER2 that might triage adenocarcinoma or adenosquamous uterine cervix cancers to a HER2-targeted radiopharmaceutical like the thorium-227 trastuzumab-linked conjugate. Prospective data would affirm whether HER2 expression would be a predictive biomarker for radiopharmaceutical treatment.

## Data availability statement

The raw data supporting the conclusions of this article will be made available by the authors, without undue reservation.

## Ethics statement

The studies involving human participants were reviewed and approved by University of Kentucky. Written informed consent for participation was not required for this study in accordance with the national legislation and the institutional requirements.

## Author contributions

All authors listed have made a substantial, direct, and intellectual contribution to the work, and approved it for publication.
